# Are competing risks models appropriate to describe implant failure?

**DOI:** 10.1080/17453674.2018.1444876

**Published:** 2018-03-09

**Authors:** Adrian Sayers, Jonathan T Evans, Michael R Whitehouse, Ashley W Blom

**Affiliations:** 1 Musculoskeletal Research Unit, Bristol Medical School, Southmead Hospital, Bristol; 2 Population Health Sciences, Bristol Medical School, Bristol; 3 National Institute for Health Research Bristol Biomedical Research Centre, University of Bristol, UK

## Abstract

**Background and purpose:**

The use of competing risks models is widely advocated in the arthroplasty literature due to a perceived bias in comparison of simple Kaplan–Meier estimates. Proponents of competing risk models in the arthroplasty literature appear to be unaware of the subtle but important differences in interpretation of net and crude failure estimated by competing risk and Kaplan–Meier methods respectively.

**Methods:**

Using a simple simulation we illustrate the differences between competing risks and Kaplan–Meier methods.

**Results:**

Competing risk and Kaplan–Meier methods estimate different survival quantities, i.e., crude and net failure respectively. Estimates of crude failure estimated using competing risk methods will be less than net failure as estimated using Kaplan–Meier methods.

**Interpretation:**

Kaplan–Meier methods are appropriate for describing implant failure, whereas crude survival estimated using competing risk methods estimates the risk of surgical revision as it depends on both implant failure and mortality. Both competing risk models and Kaplan–Meier methods are useful in arthroplasty, and both provide unbiased estimates of crude and net failure in the absence of any confounding or selection respectively. Surgeons and researchers should carefully consider whether the use of competing risks is always justified. Lower estimates of failure from competing risk models may be misleading to surgeons who are attempting to select the best implants with the lowest failure rates for their patients.

We have recently noticed a number of incidences in the arthroplasty literature of authors espousing the benefits of using competing risk models in preference to Kaplan–Meier (KM) estimates to describe the failure of implants due to a perception that the observed high mortality rates in elderly patients may lead to biased estimates using the KM method (Biau et al. 2007, Fennema and Lubsen 2010, Keurentjes et al. 2012, Lacny et al. 2015, Porcher 2015, Wongworawat et al. 2015, Martin et al. 2016, Lampropoulou-Adamidou et al. 2017). This recent trend is somewhat worrying as we believe there is a fundamental misinterpretation of what Kaplan–Meier (KM) (Kaplan and Meier 1958) or competing risks (CR) (Coviello and Boggess 2004) models estimate, and under which circumstances each method may be preferable.

To correct this misunderstanding, we describe a simple simulation in a hypothetical situation with immortal patients, where no individuals are ever lost to follow-up. Figure 1 panel (a) illustrates this process using a line plot which illustrates when a patient becomes at risk and when a failure occurs and exits the study. In this situation, it is very easy to estimate implant survival at a time of interest, i.e., it is simply the proportion of those who fail. The numerator is the number of failures, and the denominator is the number of patients implanted. A simple proportion, KM estimates (Kaplan and Meier 1958), and the cumulative incidence function (CIF) (Coviello and Boggess 2004) from a CR model will give identical estimates. This scenario is the ideal scenario, as we need not concern ourselves with problems such as censoring (loss to follow-up or mortality), and we describe these estimates of failure as net failure, using the terminology of Lambert et al. (2010).

**Figure 1.**
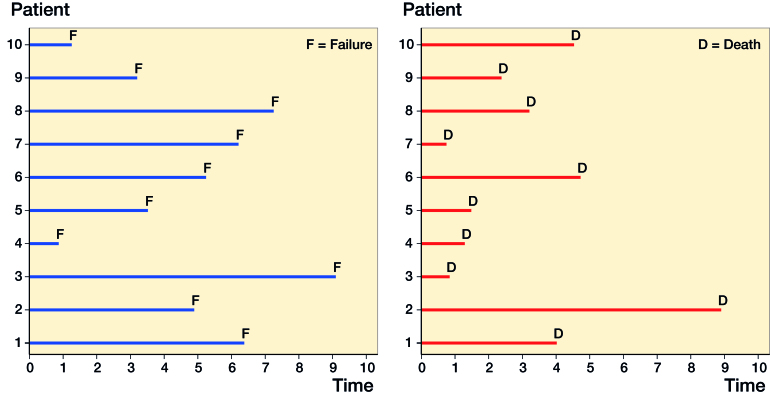
Panel (a) is a line plot that illustrates the time at risk of 10 patients entering a study following arthroplasty (time 0) and exiting the study after failure where the only possible mechanism of exiting the study is failure, i.e., no other cause of censoring occurs. Panel (b) is a line plot that illustrates a non-informative mortality profile of the same 10 patients entering a study following arthroplasty (time 0).

However, some researchers are under the misguided belief that this hypothetical situation is the only scenario in which the KM estimator is appropriate (Biau et al. 2007). The title of Kaplan and Meier’s (1958) seminal work, “Nonparametric-Estimation from Incomplete Observations,” gives us a clue to why this is incorrect. The KM method was specifically developed to allow incomplete observations due to non-informative right censoring, i.e., individuals cease to be at risk of failure, but have not failed where the reason that they cease to be at risk is completely independent of the cause of failure.

In arthroplasty failure studies, mortality is one possible cause of being censored. Figure 1 panel (b) illustrates a non-informative mortality profile of patients in Figure 1 panel (a).

In this more complex and alternate situation with mortal patients, the failure process is more difficult to estimate due to the presence of a mortality process. This additional process removes patients from the study and calculation of failure becomes more complex—see Figure 2 which overlays the failure and mortality processes.

**Figure 2.**
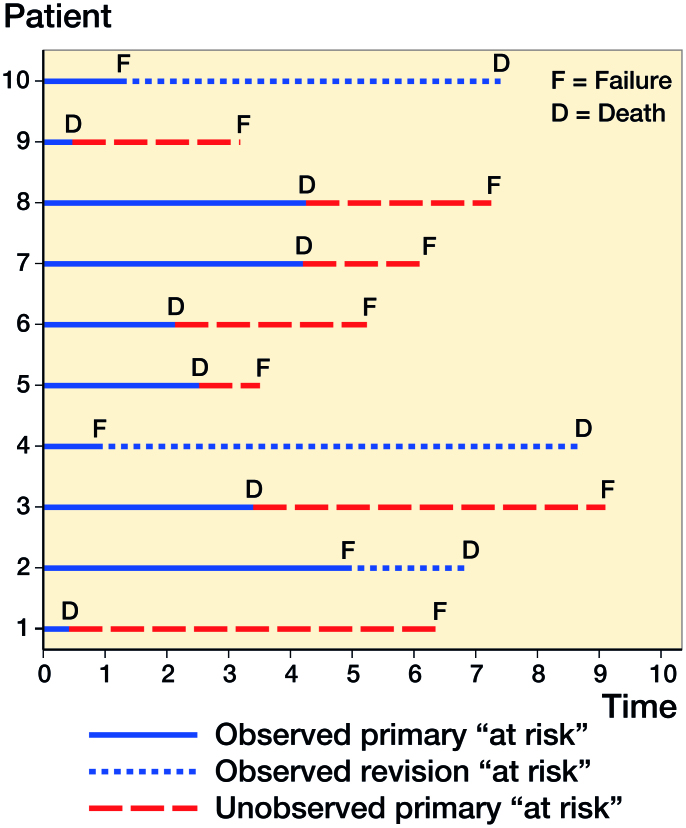
A line plot that illustrates the time at risk of 10 patients entering a study following arthroplasty (time 0) and the combination of a failure and mortality mechanism, i.e., mortal patients.

Due to the complexity of this alternate situation with mortal patients, we are confronted with a choice of what to estimate. We can attempt to recover an estimate of net failure, which gives us an estimate of the failure of the implant, i.e., the failure estimate from the immortal cohort. Or, we can estimate crude failure, which represents the likely number of failures we see in practice, i.e., it is a composite of both the failure of the implants and the mortality process. The terminology used in this field is somewhat heterogeneous, therefore we use the terminology described by Lambert et al. (2010).

Standard methods of conducting survival analysis, i.e., KM or Cox regression focus on net failure, are based solely on the hazard profile of the cause of interest. Competing risk methods estimate crude failure and depend on both the hazard of the event of interest and the hazard of the competing event.

The differences in the KM estimate with immortal patients and mortal patients and the CIF (competing risks estimate) with mortal patients is presented in Figure 3. Here, we simply create 2 independent random uniform failure profiles between 0 and 10 years for 2 processes, (1) implant failure, and (2) mortality for 1,000 patients. Analysis of implant failure of immortal patients, ignoring the mortality process, can be considered the “truth,” and removing patients from the risk set due to a mortality event creates a mortal cohort, i.e., the observed. We expect the failure to be 100% at 10 years, and a straight line from 0 years to 10 years, i.e., a 45-degree line. This clearly illustrates the CIF (competing risks estimate) is not the same as that of KM. It is a biased estimate of net failure, but an unbiased estimate of crude failure. Whilst the simulation is extreme, i.e., everyone fails and everyone dies, the results will hold in all circumstances that the censoring is non-informative. The degree to which the CIF is different from the KM profile depends on the mortality process. Prior to the first mortality event, KM and CIF are equal, and only following the first mortality event do they become unequal. In arthroplasty research differences between KM and CIF are likely to be more evident in series with long-term follow up, where mortality is inevitably higher, or in series with elderly or frail patients.

**Figure 3.**
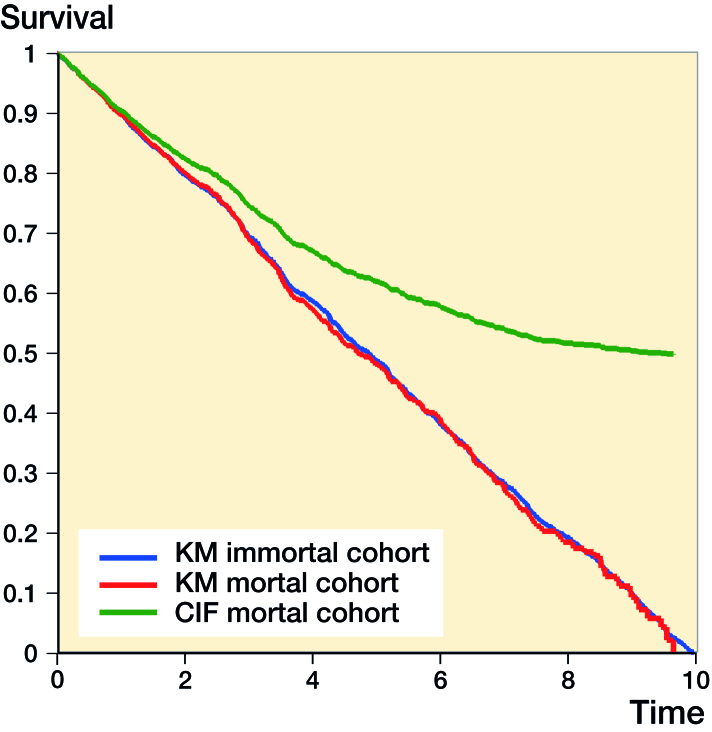
KM survival curves and the 1 minus the cumulative incidence function in mortal and immortal cohorts.

These differences are well known to those with a methodological interest in survival analysis. For example, Gooley et al. (1999) note that if one is interested in evaluating a cause-specific failure, the CIF may be misleading and inferences should be made from functions which are based solely on the hazard of failure from the cause of interest, i.e., use the KM estimator. Putter et al. (2007) similarly state that the “naive Kaplan–Meier estimator describes what would happen if the competing event could be prevented to occur, creating an imaginary world in which an individual remains at risk of failure from the event of interest,” i.e., an immortal patient cohort. Ranstam et al. (2011) describe this in an arthroplasty setting as the “implicit assumption that the patient will be alive until the implant fails.” Recently, we have similarly illustrated this result using a simulation study in the context of prosthesis benchmarking: we illustrate that KM provides unbiased estimates of net failure and provide nominal coverage, i.e., the confidence interval includes the true value on 95% of occasions (Sayers et al. 2017).

In as far as we currently know, the mortality process is independent of whether implants are revised or not, i.e., mortality satisfies the non-informative censoring assumption. Our belief in this assumption is based on the observation that even when an implant or group of implants fail in a large number of patients, e.g., metal on metal, this is not associated with any increase in pathologies, in the short term, such as cancer that in turn may lead to an excess of mortality (Smith et al. 2012a, 2012b, 2012c). However, it is important these assumptions are checked periodically; an absence of evidence is not evidence of absence, and future information may require analyses to be modified to account for an informative censoring profile.

Simply, competing risk methods and non-competing risk methods estimate different quantities, and which quantity you should use depends on your application of interest. If you are interested in describing the failure of an implant, comparing the failure rate of a group of implants, looking for outliers, i.e., from a regulatory perspective, or attempting to select an implant for use that has the greatest longevity, you need estimates of net failure (KM). If you are interested in resource planning, health economics, or communicating with patients their likely chance of experiencing a revision, estimates of crude failure (CR) are more likely to be desirable.

Just because the estimate of net implant failure is higher than crude failure does not mean they are not correct or desirable in many circumstances in arthroplasty. However, it also important to remember that whilst KM and the CIF are statistically unbiased estimates for net and crude failure respectively, they are both equally likely to display bias in the presence of confounding factors and selection effects, and simply choosing the appropriate approach is not a panacea against this immutable problem.

## Funding and conflict of interest

AS was supported by a MRC strategic skills fellowship: MRC Fellowship MR/L01226X/1. JTE was supported by the National Joint Registry of England, Wales, Northern Ireland and the Isle of Man and Royal College of Surgeons of England Fellowship.

This study was supported by the NIHR Biomedical Research Centre at the University Hospitals Bristol NHS Foundation Trust and the University of Bristol. The views expressed in this publication are those of the authors and not necessarily those of the NHS, the National Institute for Health Research, or the Department of Health.

We have no competing interests to declare.

See also Editorial in the June 2018 issue of Acta Orthopaedica.

*Acta* thanks Nicole Pratt and other anonymous reviewers for help with peer review of this study.

AS, JTE, MRW, AWB conceived the manuscript, interpreted data from simulation, and approved the final version of the manuscript. AS wrote the first draft and performed the simulation. JTE and AS reviewed the literature.
